# The 5′-phosphate enhances the DNA-binding and exonuclease activities of human mitochondrial genome maintenance exonuclease 1 (MGME1)

**DOI:** 10.1016/j.jbc.2022.102306

**Published:** 2022-08-05

**Authors:** Kathleen M. Urrutia, Wenyan Xu, Linlin Zhao

**Affiliations:** 1 Department of Chemistry, University of California, Riverside, Riverside, California, USA; 2 Environmental Toxicology Graduate Program, University of California, Riverside, Riverside, California, USA

**Keywords:** DNA damage, DNA enzyme, DNA repair, DNA turnover, mitochondrial DNA, 5′dRp, 5′-deoxyribosephosphate, BER, base excision repair, FAM, 6-fluorescein, FP, fluorescence polarization, MGME1, mitochondrial genome maintenance exonuclease 1, mtDNA, mitochondrial DNA, pol γ, polymerase γ

## Abstract

In higher eukaryotes, mitochondria play multiple roles in energy production, signaling, and biosynthesis. Mitochondria possess multiple copies of mitochondrial DNA (mtDNA), which encodes 37 genes that are essential for mitochondrial and cellular function. When mtDNA is challenged by endogenous and exogenous factors, mtDNA undergoes repair, degradation, and compensatory synthesis. mtDNA degradation is an emerging pathway in mtDNA damage response and maintenance. A key factor involved is the human mitochondrial genome maintenance exonuclease 1 (MGME1). Despite previous biochemical and functional studies, controversies exist regarding the polarity of MGME1-mediated DNA cleavage. Also, how DNA sequence may affect the activities of MGME1 remains elusive. Such information is not only fundamental to the understanding of MGME1 but critical for deciphering the mechanism of mtDNA degradation. Herein, we use quantitative assays to examine the effects of substrate structure and sequence on the DNA-binding and enzymatic activities of MGME1. We demonstrate that MGME1 binds to and cleaves from the 5′-end of single-stranded DNA substrates, especially in the presence of 5′-phosphate, which plays an important role in DNA binding and optimal cleavage by MGME1. In addition, MGME1 tolerates certain modifications at the terminal end, such as a 5′-deoxyribosephosphate intermediate formed in base excision repair. We show that MGME1 processes different sequences with varying efficiencies, with dT and dC sequences being the most and least efficiently digested, respectively. Our results provide insights into the enzymatic properties of MGME1 and a rationale for the coordination of MGME1 with the 3′–5′ exonuclease activity of DNA polymerase γ in mtDNA degradation.

Mitochondrial DNA (mtDNA) is a circular DNA molecule of 16,569 base pairs (bp), which encodes 13 protein subunits of the oxidative phosphorylation system, two rRNAs, and 22 tRNAs. mtDNA instability in forms of mutations, deletions, ablation, or depletion has been associated with a broad spectrum of human disorders and aging (1, 2, 3, 4, 5, 6). Critical to the maintenance of mtDNA genome stability are the proteins involved in mtDNA replication. Many disease-associated mutations have been identified in genes encoding these proteins, such as the mtDNA polymerase γ (pol γ) complex (*POLGA* and *POLGB*) (7, 8), the replicative helicase (*TWNK*) (9), DNA replication helicase/nuclease 2 (*DNA2*) (10), mitochondrial genome maintenance exonuclease 1 (*MGME1*) (11), and mitochondrial single-stranded DNA-binding protein (*SSBP1*) (12, 13, 14, 15, 16).

Human MGME1, a mitochondria-specific DNase, belongs to the PD−(D/E)XK phosphodiesterase superfamily (17). The superfamily includes a variety of enzymes involved in DNA and RNA cleavage. MGME1 interacts with all three components (POLG, SSBP1, and TWNK) of the minimal mitochondrial replisome (18, 19) and therefore is considered a component of the mitochondrial replication machinery (20). MGME1 has a documented role in maintaining 7S DNA (11, 17, 18, 21). Patients carrying MGME1 mutations or MGME1-depleted cells exhibit an increase in 7S DNA levels (11, 17, 21). Similarly, MGME1-knockout mice showed higher steady-state levels of 7S DNA with longer 5′ DNA ends (18). In addition, MGME1 regulates mtDNA replication and transcription termination. MGME1-knockout mice show a tissue-specific mtDNA replication stalling phenotype and an altered transcription profile (18). Furthermore, MGME1 cooperates with pol γ and the TWNK helicase in the degradation of linear mtDNA upon DNA double-strand breaks (22). In human embryonic kidney 293 cells, upon the induction of mtDNA double-strand breaks by mitochondria-targeting restriction enzymes, accumulation of linear mtDNA was observed in MGME1-knockout cells and cells containing pol γ D274A (an exonuclease-deficient variant) (22).

*In vitro*, recombinant MGME1 processes ssDNA substrates from the 5′ terminus or 3′ terminus and DNA flap substrates with a 5′ flap or 3′ flap (11, 17, 23). MGME1 also cleaves the ssDNA segment of 5′- and 3′-splayed-arm DNA substrates but pauses at the ssDNA–dsDNA junction (11). MGME1 does not have an endonucleolytic activity on a single-strand circular DNA (11, 17). The ability of MGME1 to recognize and bind to a free 5′-end or 3- end of a DNA molecule is particularly important in mtDNA degradation because these activities must be strictly regulated to prevent the formation of faulty linear mDNA species or multiple mtDNA rearrangements (22). Nonetheless, outstanding questions remain regarding the exonuclease activities of MGME1. First, controversies exist regarding the polarity of MGME1-mediated DNA digestion. Although it is noted by some researchers that MGME1 mainly degrades ssDNA substrates in a 3′–5′ direction (17) or that it processes ssDNA from either end (24), research by other groups pointed to preferential digestion of ssDNA in a 5′–3′ direction (11, 18, 21, 23). The 5′–3′ directionality appears to be consistent with the accumulation of extended 5′ ends of 7S DNA in fibroblasts from patients carrying MGME1 mutations (21) and in MGME1-knockout mice (18). Second, does MGME1 cleave different DNA sequences with similar efficiencies? These questions are not only fundamental to the understanding of MGME1 but also critical to deciphering the mechanism of mtDNA degradation in the contexts of mtDNA depletion syndromes (1) and mtDNA-mediated cell signaling (25).

To fill these knowledge gaps, we quantified the DNA-binding and exonuclease activities of MGME1 with various substrates. We used homopolymeric ssDNA substrates and random sequences to characterize the sequence preference of MGME1. Our results show that MGME1 localizes to a 5′-end under equilibrium binding conditions and prefers to cleave ssDNA in a 5′–3′ direction. The 5′-phosphate group plays an important role in DNA binding and optimal cleavage by MGME1. A clear difference in binding and enzymatic cleavage of MGME1 with ssDNA homopolymers with different sequences was observed. Collectively, this study answers several important questions regarding the biochemical properties of MGME1 and provides a rationale for the division of labor between MGME1 and pol γ in mtDNA degradation or processing replication intermediates.

## Results

### Stabilization of MGME1:DNA complexes by 5′-phosphate

**Figure 1:**
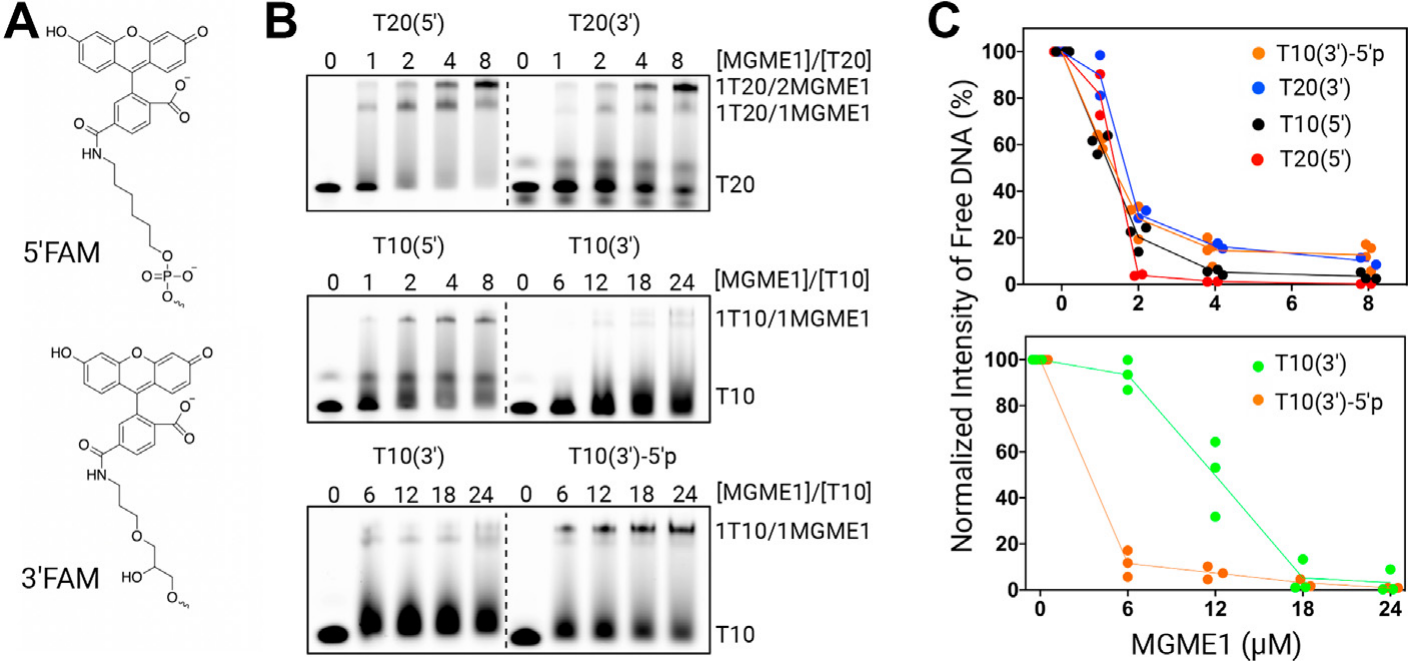
**DNA-binding stoichiometry of MGME1.***A*, structures of the FAM label at the 3′-end and 5′-end. *B*, representative gel image of EMSA demonstrating the DNA-binding stoichiometry and footprint of MGME1. *C*, quantification of the remaining unbound DNA in electrophoretic gel shift assay when different ssDNA substrates are titrated with MGME1. Errors represent SD (n = 3). FAM, 6-fluorescein; MGME1, mitochondrial genome maintenance nuclease 1.

We determined the binding stoichiometry and footprint of MGME1 on ssDNA substrates using the EMSA. Four homopolymers, poly(dT)_10_ and poly(dT)_20_ with a 5′ or 3′ 6-fluorescein (FAM) label (hereinafter referred to as T10(5′), T10(3′), T20(5′), and T20(3′)), were designed to characterize the DNA-binding activities. The use of homopolymers eliminates potential complications from any sequence effects or DNA secondary structures. The structures of 5′-FAM and 3′-FAM are shown in Figure 1*A*. As shown in Figure 1*B*, T20 can accommodate up to two MGME1 molecules (*top panel*), and T10 substrates can accommodate one MGME1 molecule (*middle panel*). These results indicate that the binding footprint is approximately 10 nt, which is in reasonable agreement with a well-defined 7-nucleotide fragment observed in the cocrystal structure of MGME1:ssDNA complexes (24). Notably, a higher yield of MGME1:DNA complexes was observed with substrates containing a 5′-FAM label (both T10 and T20). With T10(3′), a higher MGME1 concentration range had to be used to produce detectable levels of MGME1:DNA complexes. Considering that the major structural difference between the two labels lies in the 5′-phosphate group of 5′-FAM (Fig. 1*A*), we reason that MGME1 may have an intrinsic binding preference for the 5′-phosphate. To test this, we compared the MGME1-binding patterns of T10(3′) with that of T10(3′)-5′p. T10(3′) contains a 5′-OH group (the native form from solid-phase DNA synthesis), and T10(3′)-5′p contains a 5′-phosphate group. As observed in Figure 1*B* (*bottom panel*) and Fig. S1, T10(3′)-5′p produced a higher yield of MGME1:DNA complexes over the entire concentration range. Quantification of the unbound DNA substrates shows clearly that the substrates with a 5′-phosphate have less remaining ssDNA in the entire MGME1 concentration range (Fig. 1*C*). Therefore, these results demonstrate a stabilizing effect of the 5′-phosphate on MGME1:ssDNA complexes.

### Preferential interaction of MGME1 with the 5′-end

**Figure 2:**
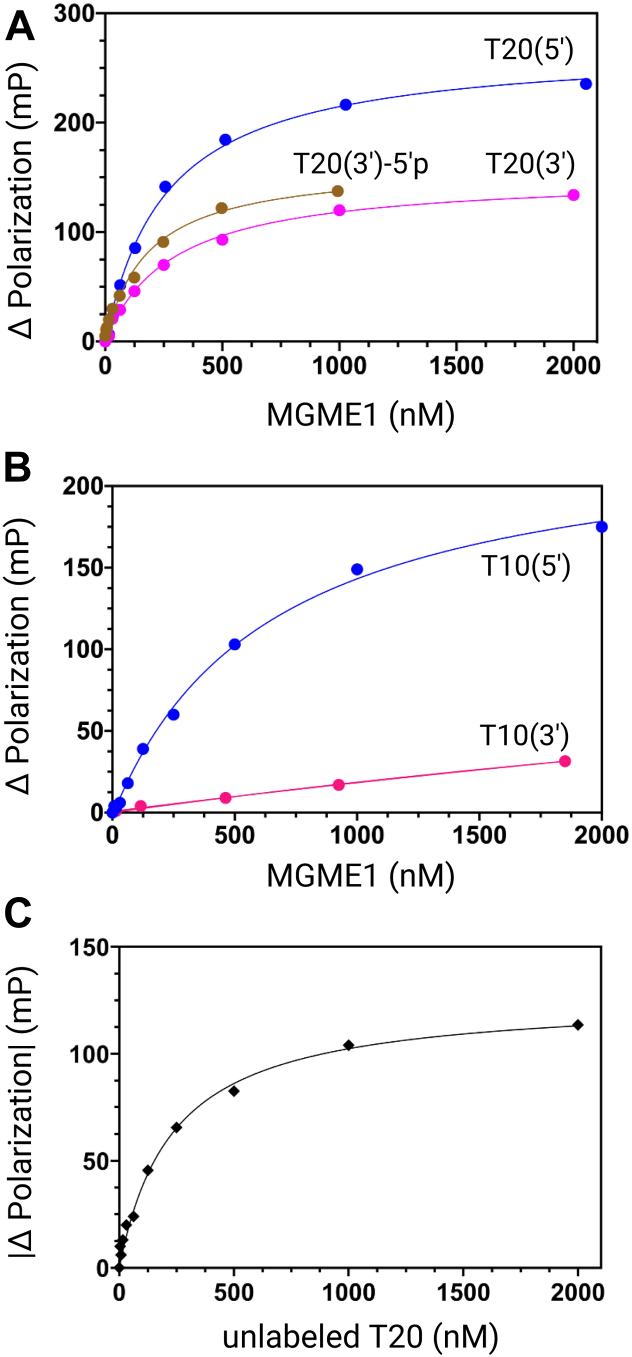
**Determination of *K***_***d*,DNA**_**of MGME1 using fluorescence polarization (FP) assays.** Data are fit to a quadratic equation (Equation 1) to obtain *K*_*d*,DNA_. *A*, representative FP changes with respective to varying concentrations of T20 substrates to obtain a *K*_*d*,DNA_ of 260 (±20) nM for T20(5′), 290 (±26) nM for T20(3′), and 179 (±24) for T20(3′)-5′p. *B*, representative FP changes with respective to varying concentrations of T10 substrates to obtain a *K*_*d*,DNA_ of 640 (±100) nM for T10(5′) and a *K*_*d*,DNA_ of 7970 (±3850) nM for T10(3′). *C*, representative FP changes with varying concentrations of an unlabeled T20 substrate. About 140 nM of MGME1 was incubated on ice with 2 nM of T20(5′) for 5 min and then titrated with increasing amounts (4–2000 nM) of T20 (unlabeled). The absolute change of polarization was used to obtain a *K*_*d*,DNA_ of 230 (±20) nM for T20 (unlabeled). Errors represent SE of the fit (Equation 1). MGME1, mitochondrial genome maintenance nuclease 1.

To compare quantitatively the binding affinity of MGME1 with different substrates, we determined the apparent equilibrium dissociation constant (*K*_*d*,DNA_) using fluorescence polarization (FP) assays. With T20, MGME1 showed an approximately 40% higher binding affinity toward T20(5′) relative to T20(3′), with a *K*_*d*,DNA_ of 240 (±36) nM for T20(5′) and a *K*_*d*,DNA_ of 330 (±83) nM for T20(3′), as shown in Figure 2*A* and Table 1. Including a 5′-phosphate group in the T20(3′) substrate resulted in a *K*_*d*,DNA_ of 180 (±5) nM for T20(3′)-5′p, consistent with the effect of 5′-phosphate increasing the stability of the MGME1:ssDNA complexes in EMSA. With T10, MGME1 exhibited an approximately 12-fold higher binding affinity toward T10(5′) relative to T10(3′) (Fig. 2*B*). Including a 5′-phosphate group in the T10(3′) substrate also led to an approximately fivefold increase in *K*_*d*,DNA_ (Table 1 and Fig. S4). The overall lower *K*_*d*,DNA_ values for T20 substrates are consistent with their larger binding platforms relative to T10 substrates. The higher affinities observed with the 5′-labeled substrate with T10 and T20 substrate sets are consistent with a preferential binding with these substrates in EMSA. The greater difference in *K*_*d*,DNA_ with the T10 substrates is likely because of ability of these substrates binding to only one MGME1 molecule. Importantly, an overall larger anisotropy change was observed with 5′-labeled substrates. Because the larger anisotropy amplitude could be due to the interaction of MGME1 with the 5′-end or the fluorescein molecule, competitive FP assays were performed using an unlabeled T20. To solutions containing preformed MGME1:T20(5′) complexes, varying concentrations of unlabeled T20 were titrated. A gradual decrease in FP signal was observed, indicative of a competitive binding by the unlabeled T20. Fitting the absolute change of FP values with respect to the T20 concentration to a hyperbolic function results in a *K*_*d*,DNA_ of 250 (±33) nM (Fig. 2*C*). The value is very similar to a *K*_*d*,DNA_ of 240 (±36) nM for T20(5′), indicating that the fluorophore contributes minimally to the binding equilibrium and that the lower *K*_*d*,DNA_ values of 5′-phosphate-containing substrates is indeed because of the presence of the phosphate group. Therefore, the overall smaller *K*_*d*,DNA_ values and greater anisotropy amplitudes with 5′ substrates, together with results from EMSA, argue for a preferential interaction of MGME1 with the 5′-phosphate.

**Table 1:** The apparent equilibrium dissociation constant (*K*_*d*,DNA_) obtained from fluorescence polarization assays

Substrates (20 nt)	*K*_*d*,DNA_ (nM)	Substrates (10 nt)	*K*_*d*,DNA_ (nM)
T20(5′)	240 ± 36	T10(5′)	600 ± 150
T20(3′)	330 ± 83	T10(3′)	7500 ± 890
T20(3′)–5′p	180 ± 5	T10(3′)–5′p	1600 ± 75
T20 (unlabeled)	250 ± 33	T10(3′)–5′dSp	340 ± 24
A20(5′)	1300 ± 170		
C20(5′)	5400 ± 130		

Reported *K*_*d*,DNA_ values are mean from two independent experiments. Errors are the range of data (n = 2).

### DNA cleavage in a 5′–3′ direction in the presence of a 5′-phosphate

**Figure 3:**
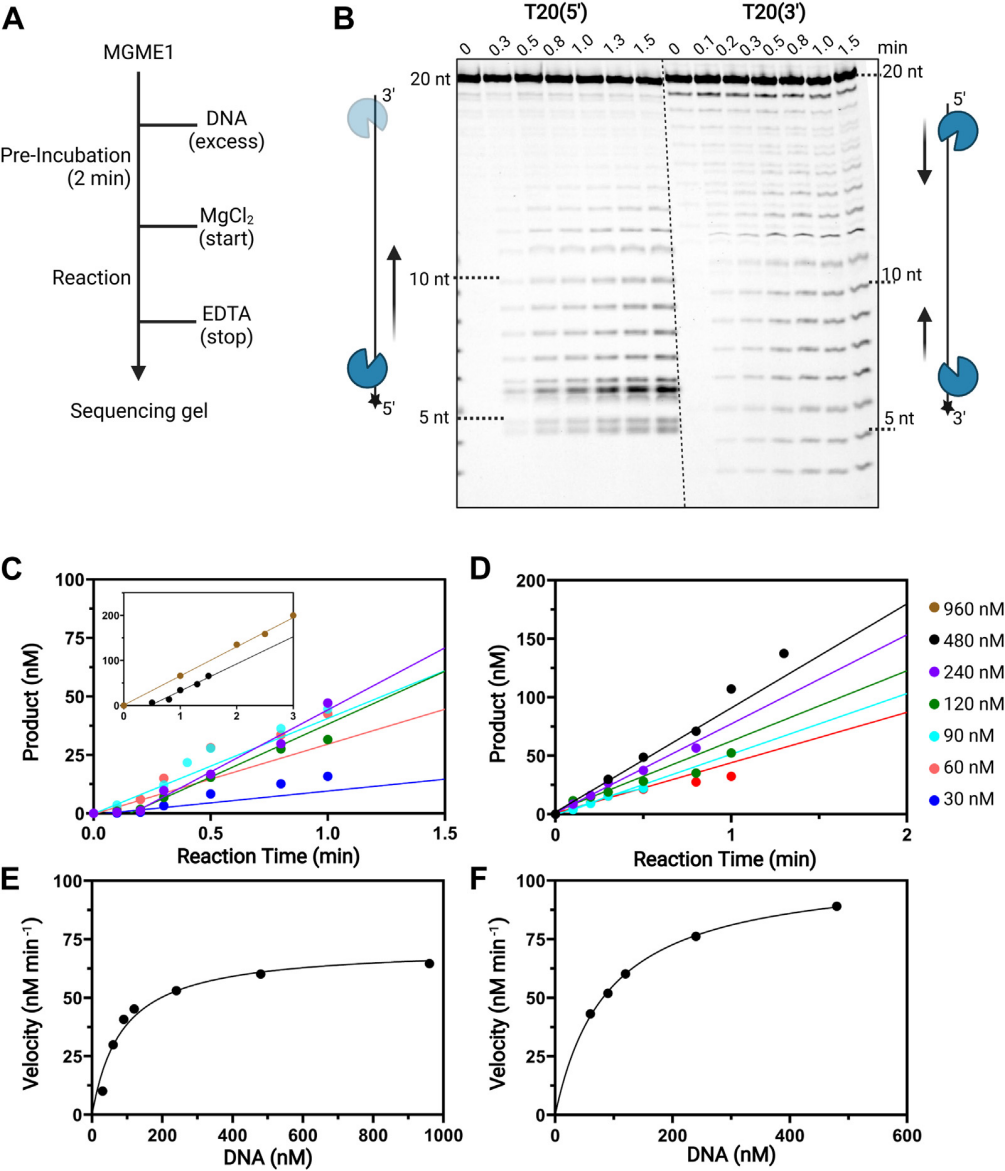
**Representative steady-state kinetic analysis of MGME1-mediated DNA cleavage.***A*, schematic illustration of the sequential mixing of different reaction components. *B*, representative denaturing PAGE analysis of digestion products under 0.48 μM ssDNA and 0.02 μM *Hs*MGME1. *Left panel*, T20(5′); *right panel*, T20(3′). Products from a reaction time course were fit to linear regression to obtain the initial velocity under each substrate concentration. *C*, T20(3′). *D*, T20(5′). The resulting velocities as a function of substrate concentration were fit to the Michaelis–Menten equation to obtain *V*_max_ and *K*_*M*_. *E*, T20(3′). *F*, T20(5′). *k*_cat_ is calculated from *V*_max_/[MGME1]. MGME1, mitochondrial genome maintenance nuclease 1.

Next, we carried out detailed kinetic analyses to quantify the exonuclease activities of MGME1. Under steady-state kinetic conditions (DNA >> MGME1), the turnover rate is contributed by all the microscopic kinetic steps and is limited by the slowest step in catalysis. Using limiting MGME1 (20 nM) and excess DNA (60–960 nM), reactions of varying times were carried out to capture the initial velocity of cleaved DNA products (Fig. 3*A*). We used the product patterns from the steady-state kinetic assays to compare qualitatively the 5′- and 3′-exonuclease activities within each substrate category. As shown in Figure 3*B* (*left panel*), MGME1 cleaves T20(5′) to yield a series of short products (5–7 nt) that appeared at earlier times with longer products appearing over time. The product pattern reflects the cleavage of T20(5′) from near the fluorophore (5′) to the other end (3′). Nearly, no products cleaved from the 3′-end were observed, in which case more products in the range of 10 to 19 nt would have formed. On the other hand, with T20(3′), MGME1 produced two sets of products. The first set of products ranged from 11 to 16 nt with shorter products accumulating over time, indicative of a 5′–3′ incision direction (digestion progressed toward the label). The second set of products (4–10 nt) showed a pattern with the intensity of longer products increased over time, indicative of a 3′–5′ cleavage direction (away from the label). These results indicate that in the presence of a 5′-phosphate, MGME1 clearly prefers a 5′–3′ incision polarity and that in the absence of the 5′-phosphate, MGME1 is able to cleave DNA from both directions.

To validate the importance of the 5′-phosphate for MGME1-catalyzed DNA cleavage, we compared the velocity of digestion with T20(3′)-5′p and T20(3′) under the identical concentration of MGME1 (20 nM, limiting enzyme). The 5′-phosphate led to a nearly fourfold higher exonuclease activity, as evidenced by initial velocities with two substrates—300 (±25) nM min^−1^ for T20(3′)-5′p and 80 (±6) nM min^−1^ for T20(3′), as shown in Fig. S2, *A* and *C*. Products from both substrates were converted to slow migration species upon phosphatase digestion (Fig. S2*B*), corresponding to the conversion of products containing a terminal phosphate to OH-containing species. Overall, compared with T20(3′), the higher velocity of cleavage with T20(3′)-5′p by MGME1 confirms the importance of the 5′-phosphate for optimal DNA digestion by MGME1.

### Efficiency of MGME1-catalyzed DNA cleavage

To compare the catalytic efficiencies quantitatively with different substrates, we obtained *k*_cat_ and *K*_*M*_ by fitting the initial velocities as a function of substrate concentration to the Michaelis–Menten equation (Fig. 3, *C*–*F* and Table 2). Despite attempts with different DNA:enzyme ratios, we could not obtain products with only one-site cleavage, consistent with the notion that MGME1 is a processive enzyme (17). We used the kinetic parameters derived from the sum of products in kinetic assays to compare substrates with a single structural variation (*e.g.*, the location of FAM, with and without 5′-phosphate) to assess the effect of substrate structure on MGME1 catalysis. With T20(3′) and T20(5′), comparable *k*_cat_, *K*_*M*_, and catalytic efficiency (*k*_cat_/*K*_*M*_) were obtained, likely because of the contribution from both 5′ and 3′ exonuclease activities, which could mask the difference in reaction rates. Considering that T10 can accommodate only one MGME1 molecule, comparing the efficiencies with T10 substrates is likely to reflect the effect of the substrate structure. Indeed, compared with T10(3′), reactions with T10(5′) yielded a comparable *k*_cat_ and a substantially lower *K*_*M*_ (Table 2 and Fig. S3). The overall catalytic efficiency with T10(5′) is fivefold higher than that of T10(3′), consistent with the stronger DNA binding observed with the 5′-labeled substrates. Notably, compared with T10(3′), a *k*_cat_ value that was approximately twofold higher was obtained with T10(3′)-5′p. Together with a comparable *K*_*M*_ value, T10(3′)-5′p resulted in an approximately twofold higher catalytic efficiency relative to T10(3′), corroborating the importance of the 5′-phosphate for optimal DNA cleavage by MGME1.

**Table 2:** Steady-state kinetic parameters of MGME1-catalyzed ssDNA cleavage

Substrates	*k*_cat_ (min^−1^)	*K*_*M*_ (nM)	*k*_cat_/*K*_*M*_ (min^−1^ nM^−1^)
T20(3′)	5.1 ± 2.1	79 ± 12	6.5 × 10^−2^
T20(5′)	4.8 ± 3.2	98 ± 17	4.9 × 10^−2^
T10(3′)	2.7 ± 1.5	216 ± 42	1.3 × 10^−2^
T10(5′)	2.8 ± 1.9	46 ± 18	6.1 × 10^−2^
T10(3′)–5′p	5.0 ± 3.3	232 ± 56	2.2 × 10^−2^

Initial velocities were obtained from a reaction time course under varying concentrations of ssDNA. The resulting velocities as a function of substrate concentration were fit to the Michaelis–Menten equation to obtain *V*_max_ and *K*_*M*_. *k*_cat_ is calculated from *V*_max_/[MGME1]. Errors represent SD (n = 3).

**Figure 4:**
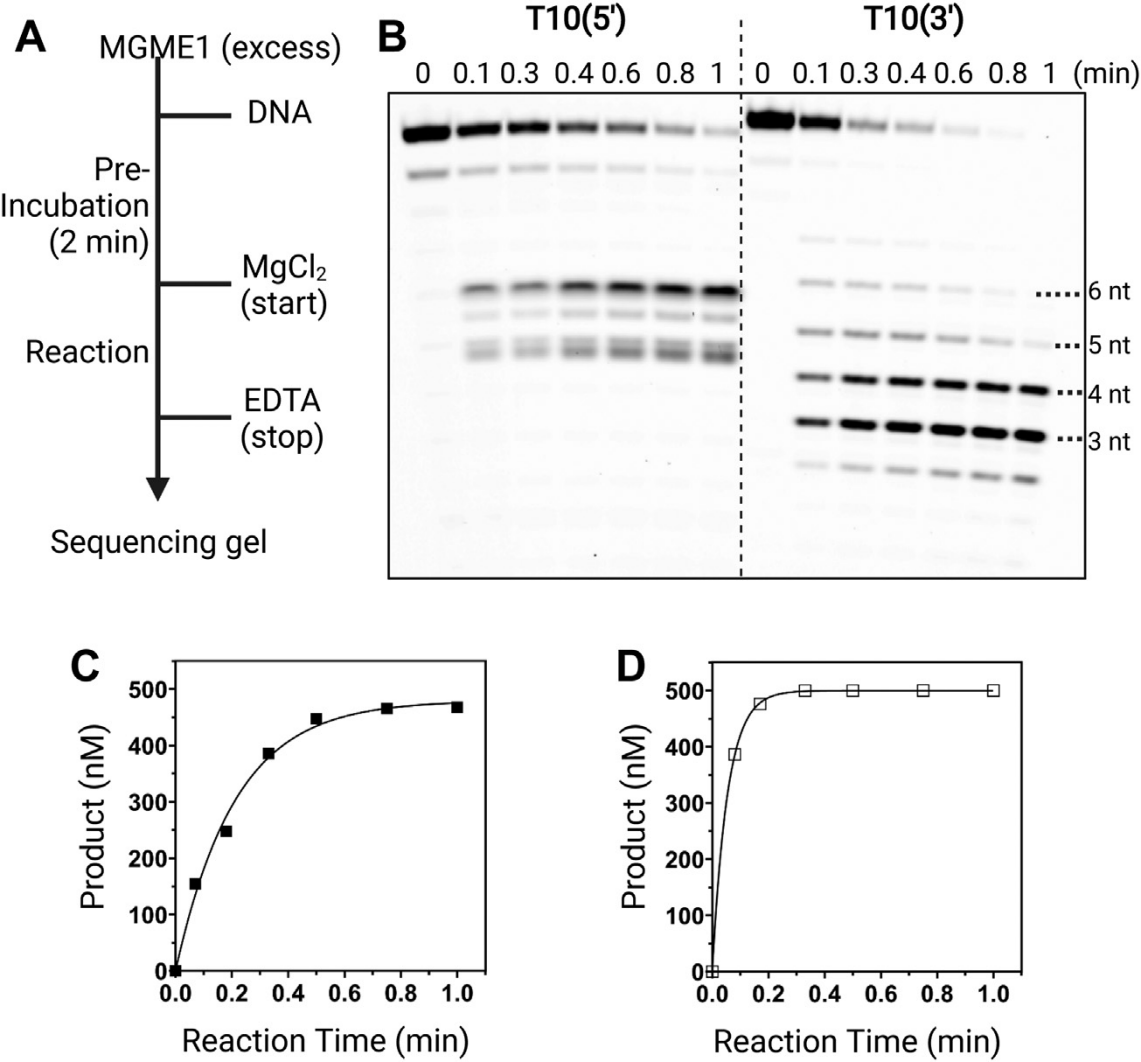
**Single-turnover kinetics of MGME1-catalyzed digestion of T10 substrates.***A*, schematic illustration of the sequential mixing of different reaction components. Assays contained 4 μM MGME1 and 0.5 μM T10. *B*, representative denaturing PAGE analysis showing different cleavage patterns from T10(5′) (*left*) and T10(3′) (*right*). Reaction products were fit to a single-exponential function (Equation 2) to obtain the apparent excision rate (*k*_exc_). *C*, T10(5′). *D*, T10(3′). MGME1, mitochondrial genome maintenance nuclease 1.

Furthermore, we obtained the excision rate (*k*_exc_) under single-turnover conditions (MGME1 > DNA) with T10 substrates. T10 was chosen to limit the complication from the association with multiple MGME1 molecules on the basis of EMSA results. For single-nucleotide excision, the single-turnover experiment captures kinetic steps up to and including the chemistry step; however, because the MGME1 processes multiple nucleotides in these reactions, *k*_exc_ encompasses additional microscopic kinetic steps such as MGME1 translocation on DNA. As shown in Figure 4*A*, a ladder-like pattern was observed with T10(3′), whereas fewer cleavage sites were observed with T10(5′), indicating that MGME1 cleaves T10(3′) in a more processive manner. The reaction time courses were fit to a single exponential equation to obtain a *k*_exc_ of 5.1 (±0.5) min^−1^ for T10(5′) and 18 (±0.4) min^−1^ for T10(3′) (Fig. 4*B* and Table 3). The overall threefold faster excision rate of T10(3′) relative to T10(5′) is somewhat surprising; however, considering that *k*_exc_ does not contain contribution from steps such as product release like the steady-state kinetic experiments, the lower *k*_exc_ the higher *k*_cat_/*K*_*M*_ for T10(5′) can be attributed to a rate-limiting step after chemistry (*e.g.*, product release) for T10(5′). The obtained *k*_exc_ values should be considered as the lower limit of the maximal excision rate because not all the substrates are saturated with MGME1 on the basis of *K*_*d*,DNA_, especially for T10(3′). The large *K*_*d*,DNA_ of T10(3′) prevented the complete saturation of the DNA substrate even at the impractical concentrations. Normalized *k*_exc_∗ values on the basis of the concentration of MGME1:T10 complexes revealed an even greater difference between T10(3′) (52 min^−1^) and T10(5′) (5.9 min^−1^). Therefore, compared with results with T10(5′), a lower catalytic efficiency with T10(3′) under steady-state conditions and a greater excision rate under single-turnover conditions are indicative of a rate-limiting step in the MGME1-mediated cleavage of T10(3′).

**Table 3:** Apparent excision rate (*k*_exc_) and the estimated maximal excision rate (*k*_exc_∗) under single-turnover conditions with T10 substrates

Substrates	*k*_exc_ (min^−1^)	[MGME1:DNA]/[DNA] (%)	*k*_exc_∗ (min^−1^)
T10(5′)	5.1 ± 0.5	86	5.9 ± 0.6
T10(3′)	18 ± 0.4	34	53 ± 1.2
T10(3′)–5′p	29 ± 2.9	69	42 ± 4.2
T10(3′)–5′dSp	22 ± 1.4	91	24 ± 1.5
R3/R2(3′)	13 ± 4		
R3/R2(3′)–5′p	24 ± 7		

Data were obtained by fitting the amount of products as a function of reaction time to a single exponential equation (Equation 2). The concentration of MGME1:DNA complexes was calculated based on a quadratic equation (Equation 3). *k*_exc_∗ is calculated by diving *k*_exc_ by the concentration of the complex. R3/R2(3′) is a dsDNA substrate containing a 10-nt 5′-overhang; R3/R2(3′)–5′p is the 5′ phosphorylated substrate. DNA sequences are shown in Table S1. Reported rates are mean from two independent experiments. Errors indicate the range of data (n = 2).

### Effect of DNA-terminal structures on MGME1 catalysis

**Figure 5:**
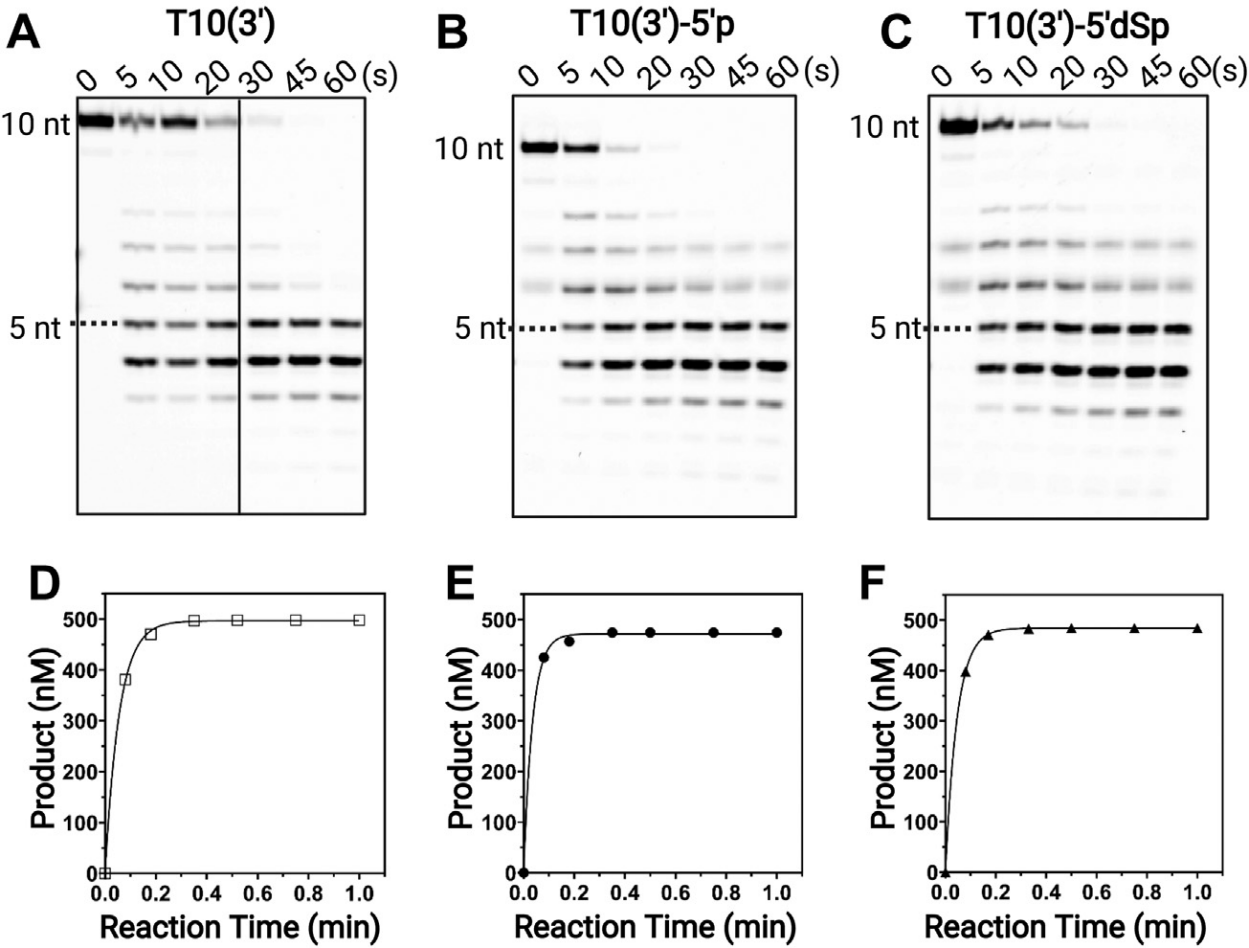
**Effect of 5′-terminal structures on MGME1 catalysis under single-turnover conditions.** Representative gel images of DNA cleavage by MGME1 with (*A*) T10(3′), (*B*) T10(3′)-5′p, or (*C*) T10(3′)-5′dSp. Fitting of the amount of products to a single-exponential function (Equation 2) allows the extraction of the excision rate (*k*_exc_) of 18 (±0.4) min^−1^ for T10(3′) in (*D*), 29 (±2.9) min^−1^ for T10(3′)-5′p in (*E*), and 22 (±1.4) min^−1^ for T10(3′)-5′SpC3 in (*F*). Reactions contained 4 μM MGME1 and 0.5 μM DNA. Errors in parenthesis are the range of data from two independent experiments (Equation 2). MGME1, mitochondrial genome maintenance nuclease 1.

To investigate the effect of 5′-phosphate and other biologically relevant terminal structures on MGME1 catalysis, we conducted single-turnover assays with T10(3′) substrates bearing different 5′-terminal structures. These substrates include T10(3′), T10(3′)-5′p, and T10(3′)-5′dSp with a 5′-phosphate group attached to a tetrahydrofuran functionality. The 5′dSp group mimics the 5′-deoxyribosephosphate (5′dRp) intermediate in base excision repair (BER), formed by apurinic/apyrimidinic endonuclease 1–mediated DNA incision at abasic sites. Previous research has suggested that 5′dRp is one of the most common BER intermediates during methylation DNA damage and subsequent repair (26). Considering its role in mtDNA degradation, MGME1 likely encounters different DNA-terminal structures. Under single-turnover conditions, the highest *k*_exc_ 29 (±2.9) min^−1^ was observed with T10(3′)-5′p, followed by 22 (±1.4) min^−1^ for T10(3′)-5′SpC3, with 18 (±0.4) min^−1^ for T10(3′) being the slowest (Fig. 5 and Table 3). These results show that a 5′-phosphate is important for the optimal exonuclease activity of MGME1. Again, the observed product patterns with longer products diminished over time confirm a 5′–3′ direction of MGME1-mediated cleavage. Notably, the overall product patterns and the *k*_exc_ values are comparable between T10(3′)-5′p and T10(3′)-5′dSp, indicating that a 5′dSp modification does not affect the efficiency of MGME1 very much. One the basis of the crystal structures of MGME1:ssDNA complexes, MGME1 cleaves the phosphoester bond 4 to 5 nucleotides away from the substrate terminus (24). Such structural arrangements could allow MGME1 to accommodate certain DNA modifications at the 5′-end, such as 5′dRp. Moreover, we evaluated the importance of 5′-phosphate in dsDNA substrates containing a 10 nt 5′-overhang. The substrates were of random sequence and contained phosphorylated and nonphosphorylated 5′-overhang and a 3′-FAM (R3/R2(3′) and R3/R2(3′)-5′p, sequences shown in Table S1). Single-turnover experiments revealed that the *k*_exc_ of R3/R2(3′)-5′p is twofold higher than that of the nonphosphorylated R3/R2(3′) substrate (Table 3 and Fig. S6), consistent with the trend observed with ssDNA substrates. Together, these results reinforce the importance of 5′-phosphate in substrate binding and catalysis by MGME1.

### Effect of the DNA sequence on the exonuclease activities of MGME1

**Figure 6:**
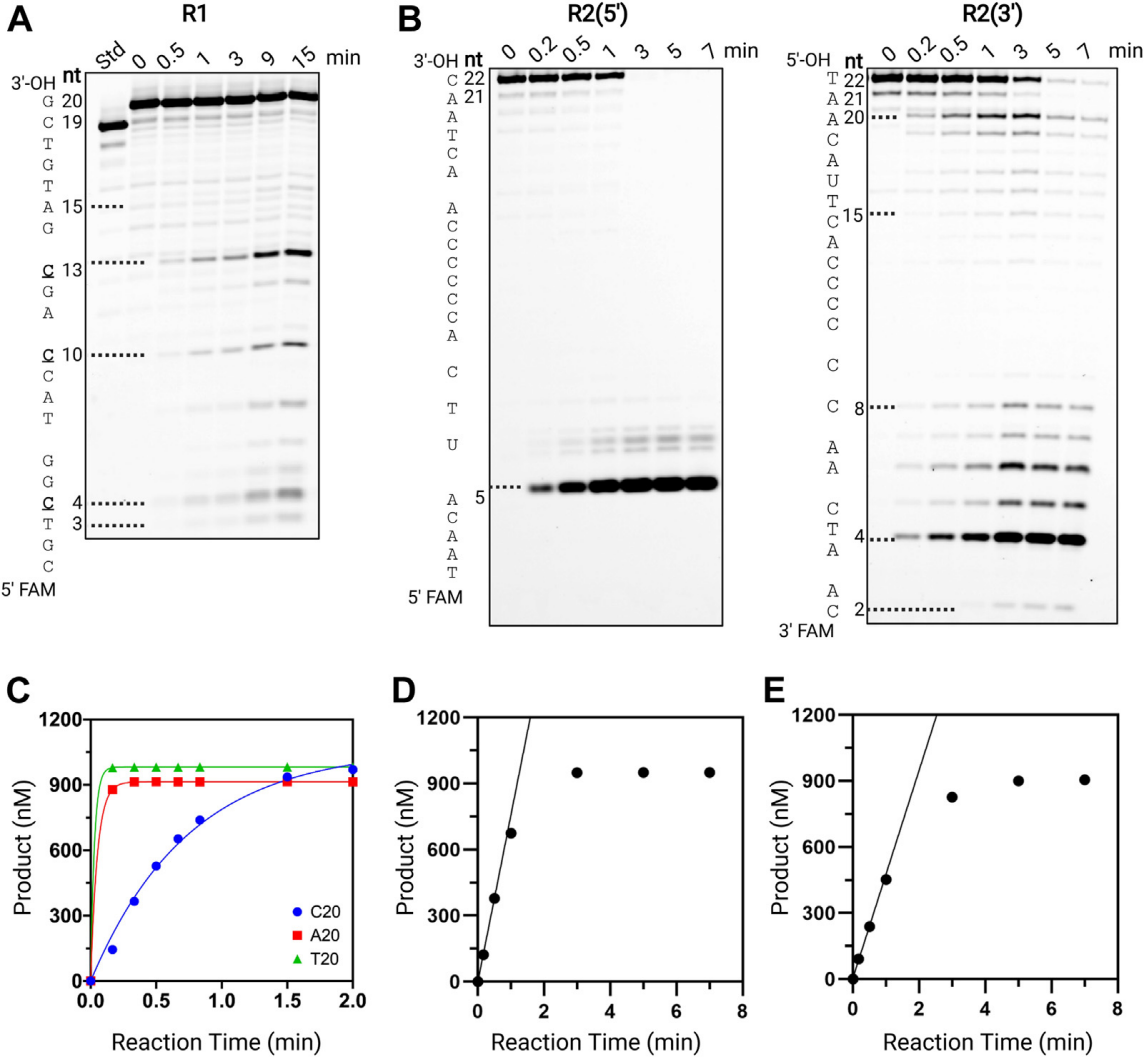
**Effect of DNA sequence on MGME1 catalysis.***A*, reaction time course of MGME1 (100 nM) with R1 substrate (500 nM). *B*, reaction time course of MGME1 (20 nM) with R2(5′) and R2(3′) substrates (1000 nM). *C*, excision of C20, A20, or T20 by MGME1 under single-turnover conditions (500 nM MGME1 and 100 nM ssDNA). Fitting the time courses to a single exponential function (Equation 2) resulted in a *k*_exc_ of 0.6 ± 0.05 min^−1^ for C20, 12 ± 1 min^−1^ for A20, and (*D*) 26 ± 1 min^−1^ for T20(5′). *D* and *E* are quantification of total products in (*B*) to obtain the initial velocities for R2(5′), 760 (±8) nM min^−1^, and for R2(3′), 470 (±26) nM min^−1^. MGME1, mitochondrial genome maintenance nuclease 1.

To examine the potential effect of DNA sequence on DNA-binding and exonuclease activities of MGME1, we assayed substrates with random DNA sequences under different conditions. Under excess substrate (a 5′-labeled 19-nt substrate, denoted as R1(5′), the sequence shown in Table S1), MGME1 showed clear pausing at dC positions (Fig. 6*A*). Similarly, with another 22-nt random sequence (R2) bearing consecutive dCs, MGME1 processed the dC-track poorly, as evidenced by the lack of products in the corresponding region on the gel (Fig. 6*B*). We compared the digestion efficiency of MGME1 with two versions of R2, one with a 5′-FAM, R2(5′), and one with a 3′-FAM, R2(3′). As shown in Figure 6*B*, MGME1 cleaved R2(5′) faster than with R2(3′), as evidenced by the complete disappearance of the substrate in 3 min with R2(5′). Relative to R2(3′), the initial velocity is 1.6-fold higher for R2(5′) (Fig. 6, *D* and *E*) because of the presence of the 5′-phosphate. To examine whether the sequence effect is sourced from a poor DNA-binding or catalytic activity, we measured *K*_*d*,DNA_ and cleavage rates under single-turnover conditions with 5′-FAM-labeled poly(dA)_20_ (denoted as A20(5′)) and poly(dC)_20_ (denoted as C20(5′)). A poly(G) substrate was not considered because of the potential of forming secondary structures. On the basis of *K*_*d*,DNA_, relative to T20(5′), MGME1 exhibited much weaker DNA-binding activities with A20(5′) and C20(5′) (Table 1 and Fig. S5), which explains in part the poor exonuclease activities with dC-containing sequences. Under single-turnover conditions, a clear difference in the cleavage rate was observed (Table 4, Figs. 6*C* and S7). The highest *k*_exc_ (29 min^−1^) was observed with T20, followed by A20 (13 min^−1^), with C20 (1.0 min^−1^) being the lowest. A nearly 30-fold difference in the excision rates of T20 and C20 substrates augment the poorly cleaved dC-containing regions with random substrates. Although poly(dC) substrates with 12 nt or longer tend to form i-motif folds, the formation of such secondary structures shows strong pH dependence and is not observed at pH 8.0 (27). Considering all our assays were performed at pH 8.0, the potential effect from secondary structures of poly(dC) on the resulting kinetic parameters is likely to be minimal. Therefore, both DNA-binding and catalytic activities account for the sequence preference for MGME1. Although MGME1 is a nonspecific exonuclease, it processes different sequences with varying efficiencies, with dT sequence and dC sequence being the most and least favored, respectively.

**Table 4:** Apparent excision rate (*k*_exc_) and the estimated maximal excision rate (*k*_exc_∗) with 20-nt homopolymers under single-turnover conditions (4 μM MGME1 and 1 μM ssDNA)

Substrates	*k*_exc_ (min^−1^)	[E:D]/[D] (%)	*k*_exc_∗ (min^−1^)
T20(5′)	29 ± 12	93	31 ± 13
A20(5′)	13 ± 11	72	18 ± 15
C20(5′)	1.0 ± 0.3	40	2.5 ± 0.8

Data were obtained by fitting the amount of products as a function of reaction time to a single exponential equation (Equation 2). The concentration of E–DNA complexes was calculated based on a quadratic equation (Equation 3). Reported rates are mean from two independent experiments. Errors indicate the range of data (n = 2).

## Discussion

**Figure 7:**
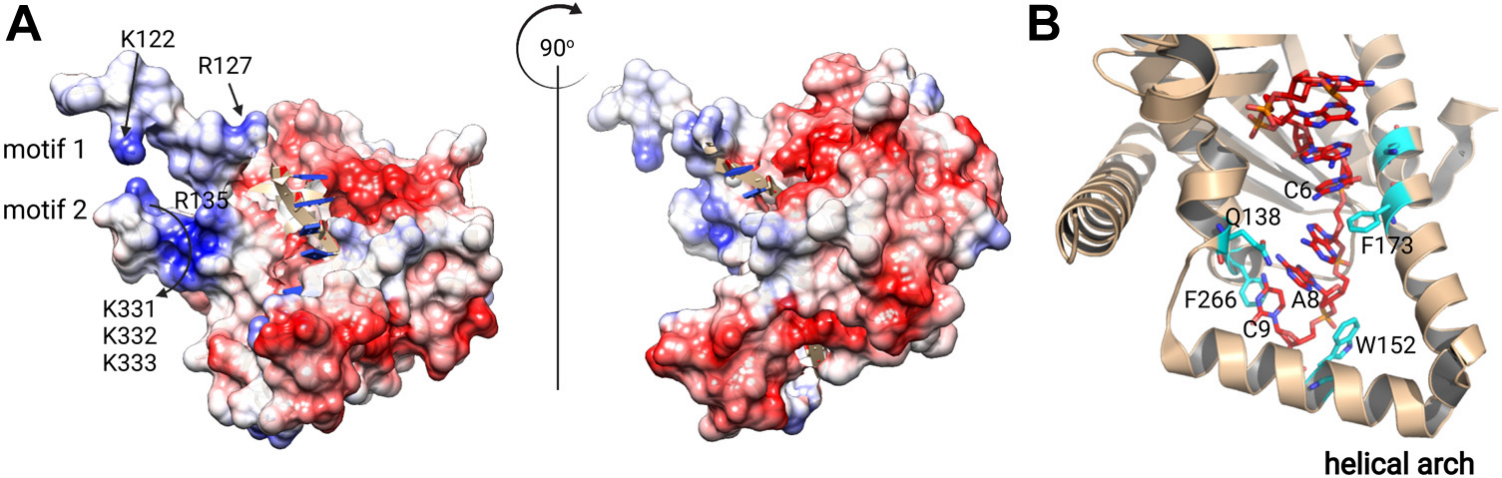
**Crystal structures of MGME1:ssDNA complex (Protein Data Bank:****5ZYU****).***A*, electrostatic surface of MGME1 with positively charged regions in *blue* and negatively charged regions in *red*. The *panel on the right* shows the side view of the same complex rotated horizontally 90° (clockwise). ssDNA is shown in *carton* with backbone in *beige*, sugar in *red*, and base in *blue*. K and R residues potentially contributing to interactions with phosphate are labeled. *B*, zoom-in view of the DNA-interacting residues in *cyan*. MGME1, mitochondrial genome maintenance nuclease 1.

MGME1 is involved in a variety of mtDNA transactions, such as the regulation of mtDNA replication, maintenance of 7S DNA, and mtDNA degradation. Although previous studies have examined the exonuclease activity of MGME1, the polarity of such activity remains controversial; whether MGME1 has any sequence preference is still unknown. Such information is fundamental to the understanding of MGME1 and also critical for gaining insights into mtDNA degradation (28), pertinent to the sentinel function of fragmented mtDNA in cellular signaling (25). In this study, we designed ssDNA substrates to probe the effect of 5′-phosphate and DNA sequence on the DNA-binding and catalytic activities of MGME1 using quantitative assays. We demonstrate unambiguously that MGME1 prefers to interact with the 5′-end of an ssDNA substrate and that a 5′-phosphate contributes to the optimal DNA binding and catalysis of MGME1. A close examination of the X-ray crystal structures of MGME1 did not show any apparent phosphate-binding pocket, unlike other 5′-exonucleases, such as human SNM1B (29). Rather, we found that several motifs could potentially mediate the interactions with phosphates on the basis of the electrostatic potential analysis (Fig. 7*A*). The first motif is at the flexible N-terminal domain, whereby several residues K122, R127, and R135 may facilitate charge–charge interactions with the phosphate, whereas other residues may interact *via* hydrogen bonding. A second motif is near the C terminus of the protein, whereby a cluster of lysine residues (K331, K332, and K333) may mediate charge–charge interactions. It is important to note that a significant portion (amino acids 21–98) of the N-terminal domain is not well defined in the solved crystal structures (24) and that the disordered region also contains a number of positively charged residues. Contributions from the positively charged residues from the region cannot be ruled out. The requirement of 5′-phosphate for optimal exonuclease activity of MGME1 also hints the potential for the positively charged motifs in facilitating translocation or product release during catalysis. Further investigation is warranted to define the roles of these motifs in terms of interaction with the terminal phosphate.

Similar to phage T5 5′-exonuclease (30), MGME1 contains a helical arch structure (Fig. 7*B*), allowing ssDNA to thread through the structure. This structural feature, together with the observation that MGME1 does not digest single-stranded circular DNA (11, 17), points to a threading mechanism for MGME1. Such a mechanism would explain why MGME1 is able to load from a 5′-end or 3′-end on the basis of our observations and reported results with 5′-flap and 3′-flap substrates (11, 23). Other 5′ nucleases that adopt a threading mechanism include FEN1 (31), albeit with a different substrate preference. The overall higher catalytic efficiencies of MGME1 observed with 5′-phosphate-containing substrates in steady-state kinetic assays argue for a preference for the 5′-end, especially in the presence of a 5′-phosphate. The observation is consistent with previous reports showing that blocking the 5′-end of an ssDNA substrate with a biotin–streptavidin complex reduced significantly (but not abolished) the exonuclease activity of MGME1, whereas such effects were not observed with a blocked 3′-end (11, 23). Considering its relatively weak DNA-binding activity, MGME1 is unlikely to be able to compete with pol γ for the 3′-end at least at the primer–template junction. The preference for a 5′-phosphate may be an important factor in regulating the activity of MGME1 to cooperate with the 3′–5′ exonuclease activity of pol γ in degrading linear mtDNA (32). In addition, MGME1 has also been proposed to remove flaps formed when mtDNA replication is reaching completion (20, 23). The preferential processing of flaps from the 5′-end would facilitate the formation of ligatable ends, which are necessary for the completion of mtDNA replication.

Although MGME1 is a nonspecific exonuclease, it shows clear binding and catalytic preferences for poly(T) homopolymers, implying that certain mtDNA sequences may be poor substrates for MGME1 and are likely to accumulate during mtDNA degradation. According to the crystal structure of MGME1 bound to an ssDNA substrate (24), the interaction with DNA is mediated by two major classes of interactions. First, interactions with the phosphate backbone are facilitated by charge–charge interactions and hydrogen bonds. These interactions are unlikely to confer any sequence specificity of MGME1. Second, several stacking interactions exist, including π–π interactions between F173 and A5 and between F266 and A8 and sugar–π interactions between W152 and the sugar pucker of A7 (Fig. 7*B*). The stacking interactions with bases are more likely to contribute to the preference for T sequences in DNA binding and catalysis. This is because, based on quantum chemical calculations, T or G generally leads to stronger interactions than A or C, with an approximately 10 kJ mol^−1^ difference in binding energy (33). Also, hydrogen bonding between Q138 and A8 may also contribute to the sequence effect (Fig. 7*B*).

According to the crystal structure, the active site of MGME1 is approximately 5 nt away from the 5′-end of the ssDNA. Such structural properties provide an explanation for the ability of MGME1 to accommodate terminal modifications, such as a fluorophore or a 5′dSp modification. The property may allow MGME1 to circumvent DNA lesions or repair intermediates at the terminus, an advantage during mtDNA degradation or removal of flaps. Similar structural arrangements have also been observed with another human mitochondrial nuclease EXOG, which is thought to process the 5′-ends in long-patch BER (34).

In summary, we demonstrate that MGME1 favors a 5′-phosphorylated DNA terminus and a 5′–3′ cleavage polarity using quantitative DNA-binding and enzyme kinetic assays. These findings resolve controversies regarding the directionality of MGME1-catalyzed DNA cleavage. A 5′-phosphate facilitates DNA-binding and exonuclease activities of MGME1, potentially mediated by a number of positively charged motifs in MGME1. MGME1 exhibits sequence preferences when processing ssDNA substrates with the highest efficiency observed for dT substrates and lowest for dC substrates. Collectively, our results provide a plausible explanation for the division of labor between MGME1 and pol γ in mtDNA degradation or processing replication intermediates. Together with other functional studies of MGME1, our data lay the foundation for a deeper understanding of the sentinel role of mtDNA in cellular signaling.

## Experimental procedures

### Reagents

Unless specified otherwise, all chemicals were of the highest quality available, purchased from Fisher Scientific or Research Products International. Modified and unmodified oligodeoxynucleotides were from Integrated DNA Technologies and were desalting or HPLC grade. *Escherichia coli* BL21 (DE3) competent cells used for expression of the wildtype human MGME1 protein were from New England Biolabs. The pET28a(+) vector expressing the DNA sequence for SUMO-*MGME1* with N-terminal SUMO-tag by NdeI/XhoI insertion was constructed based on a previous report (24).

### Expression and purification of human MGME1

MGME1 was expressed and purified based on a reported procedure with modifications. Specifically, overexpression was induced by 0.1 mM IPTG when an absorbance reached 0.6 at 600 nm and continued at 18 °C for 16 h. For purification, cells were resuspended in nickel affinity buffer (buffer A, 20 mM Tris–HCl, pH 8.0, 500 mM NaCl, 25 mM imidazole, and 5 mM beta-mercaptoethanol) supplemented with EDTA-free protease inhibitor minitablets (Thermo Fisher Scientific), and lysed using a Dounce homogenizer followed by sonication. After clarification by centrifugation, the lysate was loaded onto a HisTrap HP column (Cytiva) equilibrated with nickel buffer A. The His-SUMO-*MGME1* fusion protein was eluted from the column with nickel buffer B (20 mM Tris–HCl, pH 8.0 at 4 °C, 500 mM NaCl, 500 mM imidazole, and 5 mM β-mercaptoethanol) under an elution gradient. Fractions containing MGME1 were pooled and dialyzed against the Ulp1 buffer (50 mM Tris–HCl [pH 7.5], 100 mM NaCl, 10% [v/v] glycerol, and 1 mM DTT) for 14 to 16 h at 4 °C. The digestion by Ulp1 SUMO protease (Enzymax LLC) was allowed for 18 h at 4 °C. The sample was loaded onto the HisTrap HP column again, where the digested MGME1 without a SUMO tag was collected in the flow-through. The sample was dialyzed at 4 °C in two steps from 500 mM to 200 and 200 to 20 mM NaCl to prepare it for anion exchange where the final concentration was 20 mM NaCl, in 20 mM Hepes (pH 8), with 1 mM DTT, then loaded onto a HiTrap Q HP column equilibrated with Q buffer A (20 mM Hepes [pH 8], 20 mM NaCl, and 1 mM DTT). The final fractions were isolated from an elution gradient applied with Q buffer B (20 mM Hepes [pH 8], 1 M NaCl, and 1 mM DTT) and then exchanged into a storage buffer (20 mM Hepes [pH 8], 100 mM NaCl, 20% [v/v] glycerol, and 1 mM DTT) before flash-freezing in liquid nitrogen for storage at −80 °C. Recombinant MGME1 was purified to homogeneity with >99% purity based on SDS gel electrophoretic analysis (Fig. 1*A*). The protein concentration was determined based on UV absorbance at 280 nm and an extinction coefficient of 48,360 M^−1^ cm^−1^.

### FP assay

To characterize the DNA-binding activity of MGME1, 2 nM of FAM-labeled ssDNA was incubated with varying concentrations of MGME1 in a buffer comprised of 20 mM Hepes (pH 8.0) at 37 °C, 100 mM NaCl, 0.1 mg/ml bovine serum albumin, 1 mM DTT, and 10 mM EDTA. All components were prepared on ice and mixed onto a 96-well microplate at room temperature. The solution was equilibrated for 15 min, and FP was measured on a BioTek Synergy H1 plate reader using an excitation and emission wavelength of 485 and 528 nM, respectively. The competitive binding affinity assay was performed under the same conditions, except that varying concentrations of unlabeled T20 were added to preformed MGME1:T20(5′) complexes. The complexes were formed by mixing with 140 nM MGME1 and 2 nM T20(5′) on the basis of a range of 50 to 80% of the FP signal change observed with T20(5′). Data were fit to a quadratic equation to obtain the apparent equilibrium binding constant (*K*_*d*,DNA_).

(1) $$y=F+\frac{D\times \left(P-F\right)\times \left[K_{d,\text{DNA}}+x+D-\sqrt{{\left(K_{d,\text{DNA}}+x+D\right)}^{2}-4Dx}\right]}{2}$$

where *y* is FP, *x* is the concentration of MGME1, *D* is the concentration of DNA, *P* is the maximal polarization, and *F* is the initial polarization.

### EMSA

A series of 9 μl solutions containing 20 mM Hepes (pH 8.0), 100 mM NaCl, 1 mM DTT, 10 mM EDTA, 1 μM ssDNA, and varying concentrations of MGME1 were assembled on ice and allowed for equilibration at room temperature for 15 min. Each aliquot was mixed with 1 μl of 10× loading buffer (10 mM Hepes [pH 8.0], 40% [v/v] glycerol) and then resolved on a 1.5 mm × 7.5 cm × 10 cm 5% native 0.35× Tris/borate/EDTA-PAGE (1/55, acrylamide/bisacrylamide, 1× Tris/borate/EDTA: 89 mM Tris borate, 2 mM EDTA) at 140 V for 40 min at 4 °C.

### Steady-state kinetics

All reactions were carried out in a buffer containing 20 mM Hepes (pH 8.0) at 37 °C, 100 mM NaCl, 0.1 mg/ml bovine serum albumin, and 1 mM DTT. All components were assembled on ice. For each reaction, the reaction mixture was pre-equilibrated for 5 min on ice and then equilibrated for 2 min at 37 °C before initiating the reaction with 5 mM MgCl_2_. All listed concentrations are the final concentrations. At specific time points, an aliquot of the reaction was removed and quenched with a solution containing 95% (v/v) formamide, 50 mM EDTA, 0.025% (w/v) bromophenol blue, and 0.025% (w/v) xylene cyanol. Samples were analyzed on a 16% denaturing PAGE gel containing 7 M urea. Gels were imaged on an Amersham Typhoon RGB imager (Cytiva) and quantified using the ImageQuant software (Cytiva). The data were analyzed using GraphPad Prism (version 6.0; GraphPad Software, Inc). Initial velocities at varying concentrations were obtained by fitting the product intensities to linear regression. The obtained *k*_obs_ were fit to the Michaelis–Menten equation to obtain *k*_cat_ and *K*_*M*_.

### Single-turnover kinetics

Single-turnover assays were carried out with 4 μM *MGME1* to 0.5 μM DNA. The reaction mixture was prepared on ice in the same buffer as in steady-state kinetic assays and preincubated on ice for 5 min before use. The reaction was equilibrated for 2 min at 37 °C and then initiated with 5 mM MgCl_2_. At specific time points, 1.5 μl of the reaction mixture was withdrawn and quenched 1:5 (v/v) in a quench solution. Data were fit to a single-exponential equation,

(2) $$\left[\text{product}\right]=A\;\left(1-{\text{e}}^{-k_{\text{obs}}\;t}\right)$$

where *A* is the product amplitude, and *t* is the reaction time.

### Calculation of MGME1:DNA complex concentration

The concentration of MGME1:DNA complex was calculated on the basis of *K*_*d*,DNA_ using a quadratic equation (35)

(3) $$\left[E\cdot D\right]=\frac{K_{d,\text{DNA}}+\left[E_{0}\right]+\left[D_{0}\right]-\sqrt{{\left(K_{d,\text{DNA}}+\left[E_{0}\right]+\left[D_{0}\right]\right)}^{2}-4\times \left[E_{0}\right]\times \left[D_{0}\right]}}{2}$$

where [*E*_0_] is the enzyme concentration, [*D*_0_] is the DNA concentration, and *K*_*d*,DNA_ is the equilibrium dissociation constant.

## Data availability

All data are contained within the article and supporting information.

## Supporting information

This article contains supporting information.

## Conflict of interest

The authors declare that they have no conflicts of interest with the contents of this article.
