# Supplementary material for: The 5′-phosphate enhances the DNA-binding and exonuclease activities of human mitochondrial genome maintenance exonuclease 1 (MGME1)
Source: J Biol Chem. 2022 Aug 5;298(9):102306. doi: 10.1016/j.jbc.2022.102306 (PMC9460513; doi:10.1016/j.jbc.2022.102306)

Supporting Information

The 5′-phosphate enhances the DNA-binding and exonuclease activities of human mitochondrial genome maintenance exonuclease 1 (MGME1)

Kathleen M. Urrutia[1], Wenyan Xu[1] and Linlin Zhao[1,2,*]

[1] Department of Chemistry, University of California, Riverside, Riverside, California, 92521, United States

[2] Environmental Toxicology Graduate Program, University of California, Riverside, Riverside, Cali-fornia, 92521, United States

* To whom correspondence should be addressed. Tel: 1-951-827-9081; Email: linlin.zhao@ucr.edu

Table of Contents

Table S1. Oligodeoxyribosenucleotide sequences used in this study.

Figure S1. Purity of MGME1 and comparison of DNA-binding properties of MGME1 with T10(5′) and T10(3′)-5′p using EMSA.

Figure S2. Comparison of T20(3′) and T20(3′)-5′p degradation under enzyme-limiting conditions.

Figure S3. Steady-state kinetic assays with T10 substrates.

Figure S4. $K_{d,DNA}$ of MGME1 with T10 substrates from fluorescence polarization assays.

Figure S5. $K_{d,DNA}$ of MGME1 with A20 and C20 substrates from fluorescence polarization assays.

Figure S6. Single-turnover assays with 5′-overhang substrates.

Figure S7. Single turnover assays with homopolymeric substrates.

Supplementary Tables

Table S1. Oligodeoxyribosenucleotide sequences used in this study.

Name	Sequence
C20(5′)	5′-/6FAM/CCCCCCCCCCCCCCCCCCCC-3′
A20(5′)	5′-/6FAM/AAAAAAAAAAAAAAAAAAAA-3′
T20(5′)	5′-/6FAM/TTTTTTTTTTTTTTTTTTTT-3′
T20(3′)	5′-TTTTTTTTTTTTTTTTTTTT/6FAM/-3′
T20(3′)-5′p	5′-phosphate-TTTTTTTTTTTTTTTTTTTT/6FAM/-3′
T20	5′-TTTTTTTTTTTTTTTTTTTT-3′
T10(5′)	5′-/6FAM/TTTTTTTTTT-3′
T10(3′)	5′-TTTTTTTTTT/6FAM/-3′
T10(3′)-5′p	5′-phosphate-TTTTTTTTTT/6FAM/-3′
T10(3')-5'dSp	5′-phosphate-tetrahydrofuran-TTTTTTTTTT/6FAM/-3′
R1(5′)	5′-/6FAM/CGTCGGTACCAGCGATGTCG-3′
R2(5′)	5′-/6-FAM/TAACAUTCACCCCCCAACTAAC-3′
R2(3′)	5′-TAACAUTCACCCCCCAACTAAC/6-FAM/-3′
R2(3′)-5′p	5′-phosphate-TAACAUTCACCCCCCAACTAAC/6-FAM/-3′
R3	5′-GTTAGTTGGGGG

Supplementary Figures

Figure S1. (A) The purity (>99%) of MGME1 is shown on a 6-13% gradient SDS-PAGE. (B) Comparison of DNA-binding properties of MGME1 with T10(5′) and T10(3′)-5′p using EMSA.

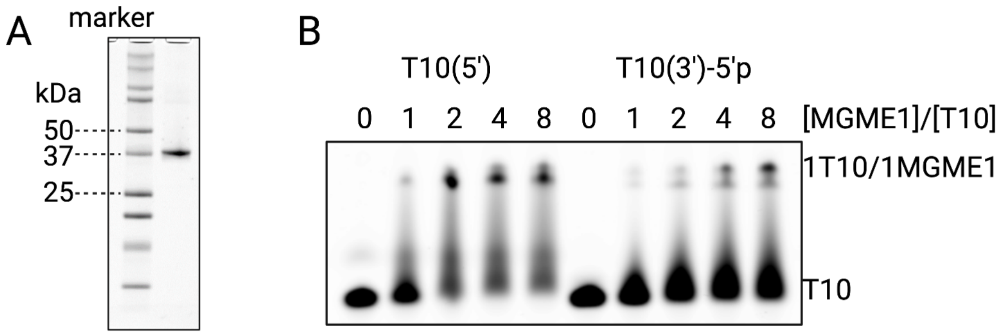

Figure S2. Comparison of T20(3′) and T20(3′)-5′p degradation under enzyme-limiting conditions. 480 nM of DNA was incubated with 20 nM of MGME1 for the appointed times. (A) 16% denaturing PAGE indicating products formed over a 1-minute time course. (B) 16% denaturing PAGE representing products formed over a 1.5 and 3 minute time interval before and after the addition of recombinant shrimp alkaline phosphatase (New England Biolabs). Splice borders indicate different parts of the gel image were reorganized to remove irrelevant lanes and to match the sample loading sequence in the labels. (C) Fitting the time course in (A) to linear regression to obtain an initial velocity of 300 (± 25) nM min^{-1} for T20(3′)-5′p (left) and 80 (± 6) nM min^{-1} for T20(3′) (right).

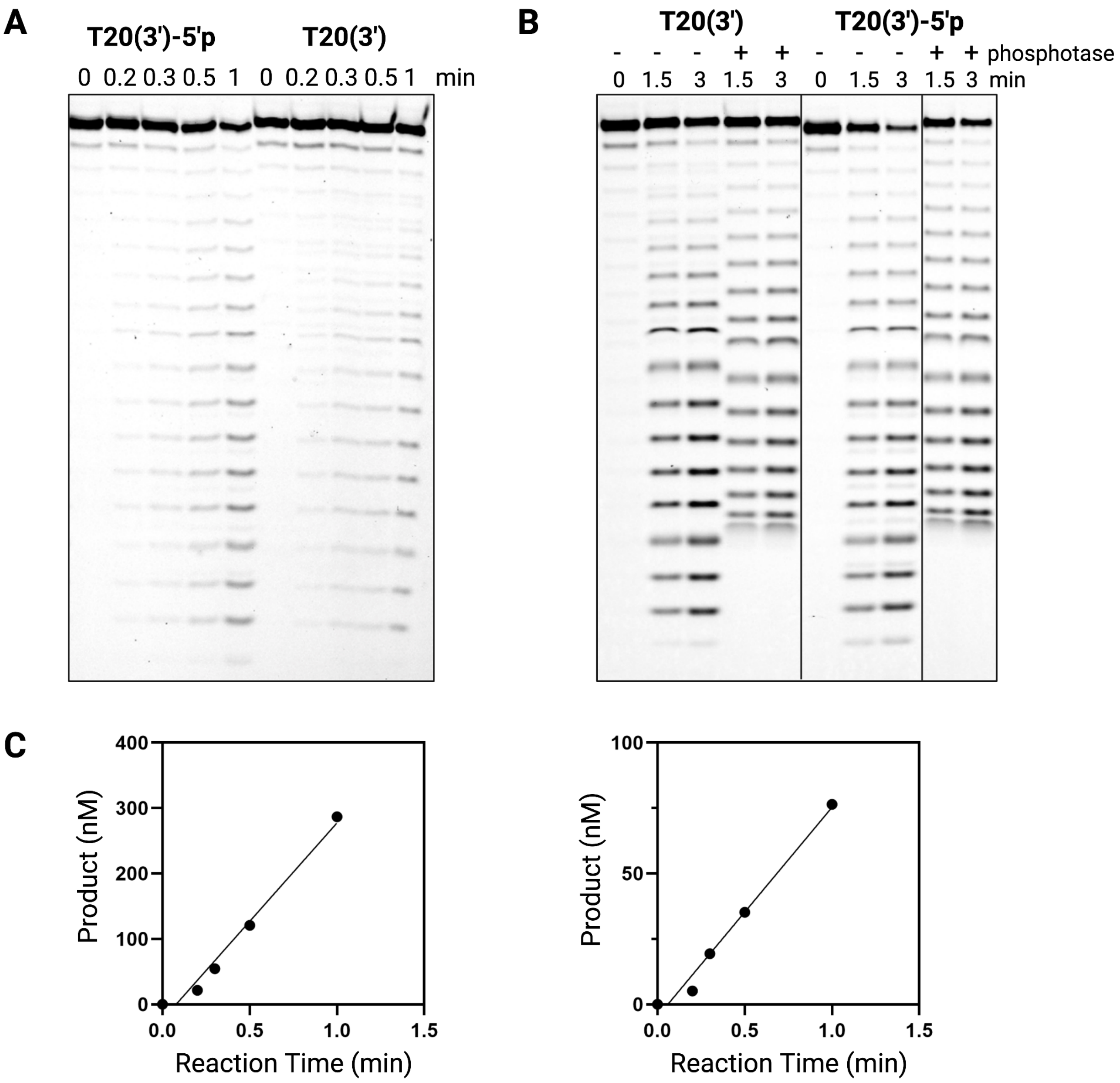

Figure S3. Steady-state kinetic assays with T10 substrates. (A), (B), and (C) representative gel images for reaction time courses with MGME1 in the presence of 120 nM of T10(5') and 120 nM T10(3'), or 240 nM of T10(3')-5'p. (D) and (E) Velocity extrapolation and fitting to the Michaelis-Menten equation for T10(5'). (F) and (G) Velocity extrapolation and fitting to the Michaelis-Menten equation for T10(3'). (H) and (I) Velocity extrapolation and fitting to the Michaelis-Menten equation for T10(3')-5'p.

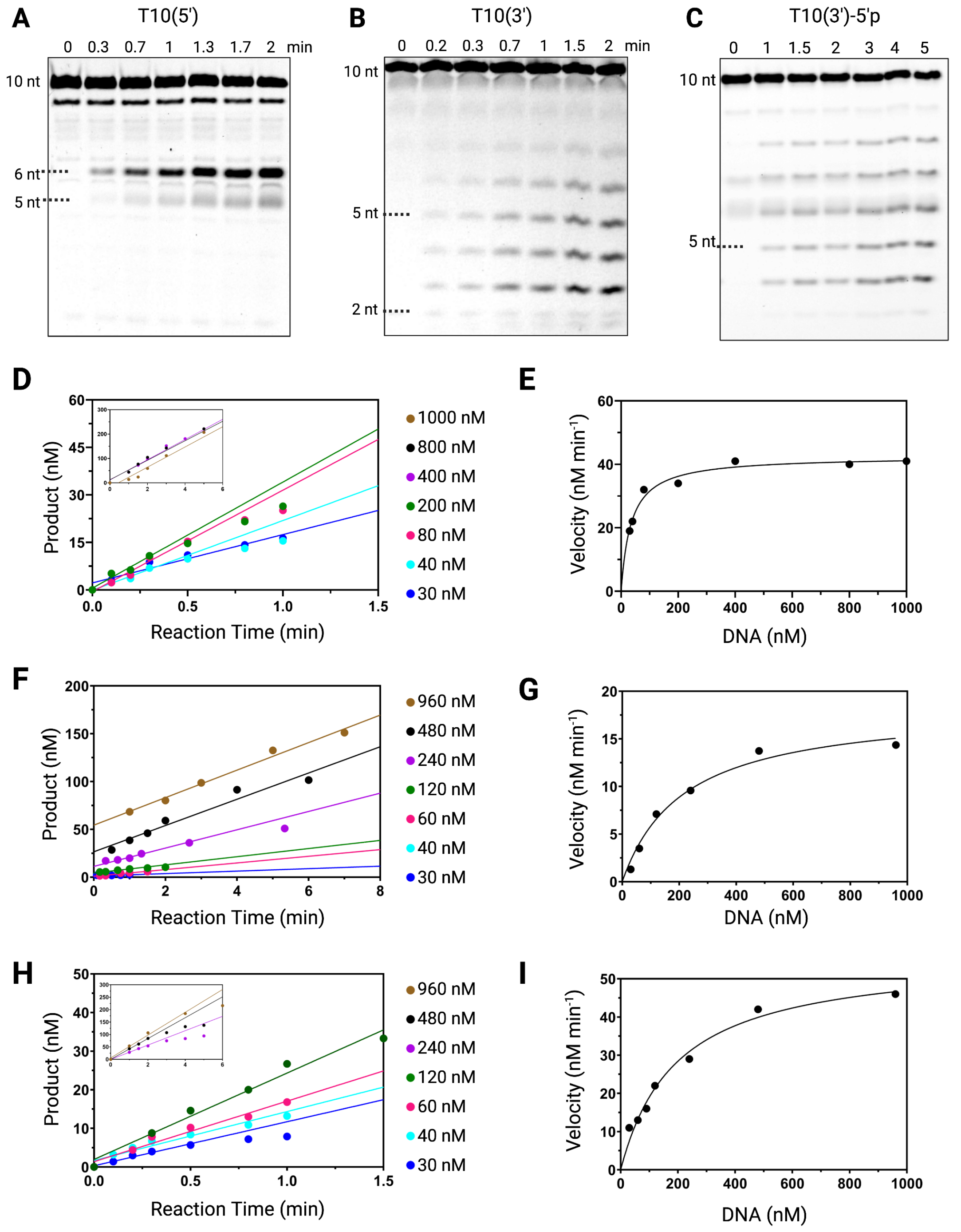

Figure S4. $K_{d,DNA}$ of MGME1 with T10 substrates from representative fluorescence polarization assays. $K_{d,DNA}$ values are summarized in Table 1.

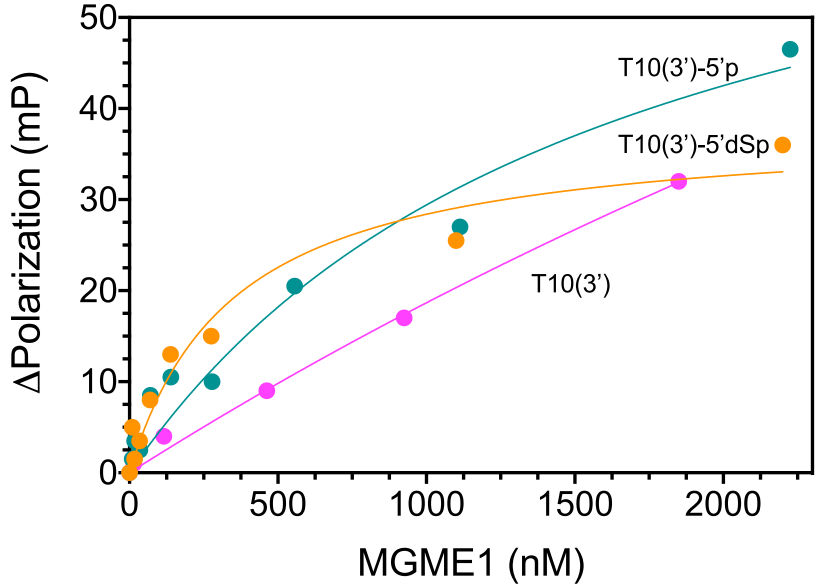

Figure S5. $K_{d,DNA}$ of MGME1 with C20 (left) and A20 (right) substrates from representative fluorescence polarization assays. $K_{d,DNA}$ values are summarized in Table 1.

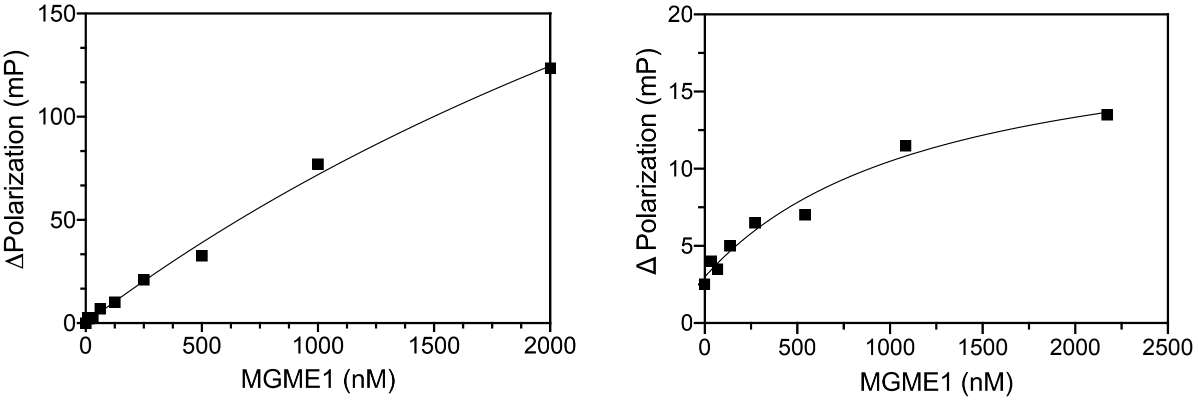

Figure S6. Single-turnover assays with 5′-overhang substrates, prepared by annealing oligomers R2(3′) or R2(3′)-5′p with R3 shown in Table S1. (A) Representative gel image. (B) Excision rates (summarized in Table 3) obtained by fitting the amount of products as a function of time to a single exponential equation.

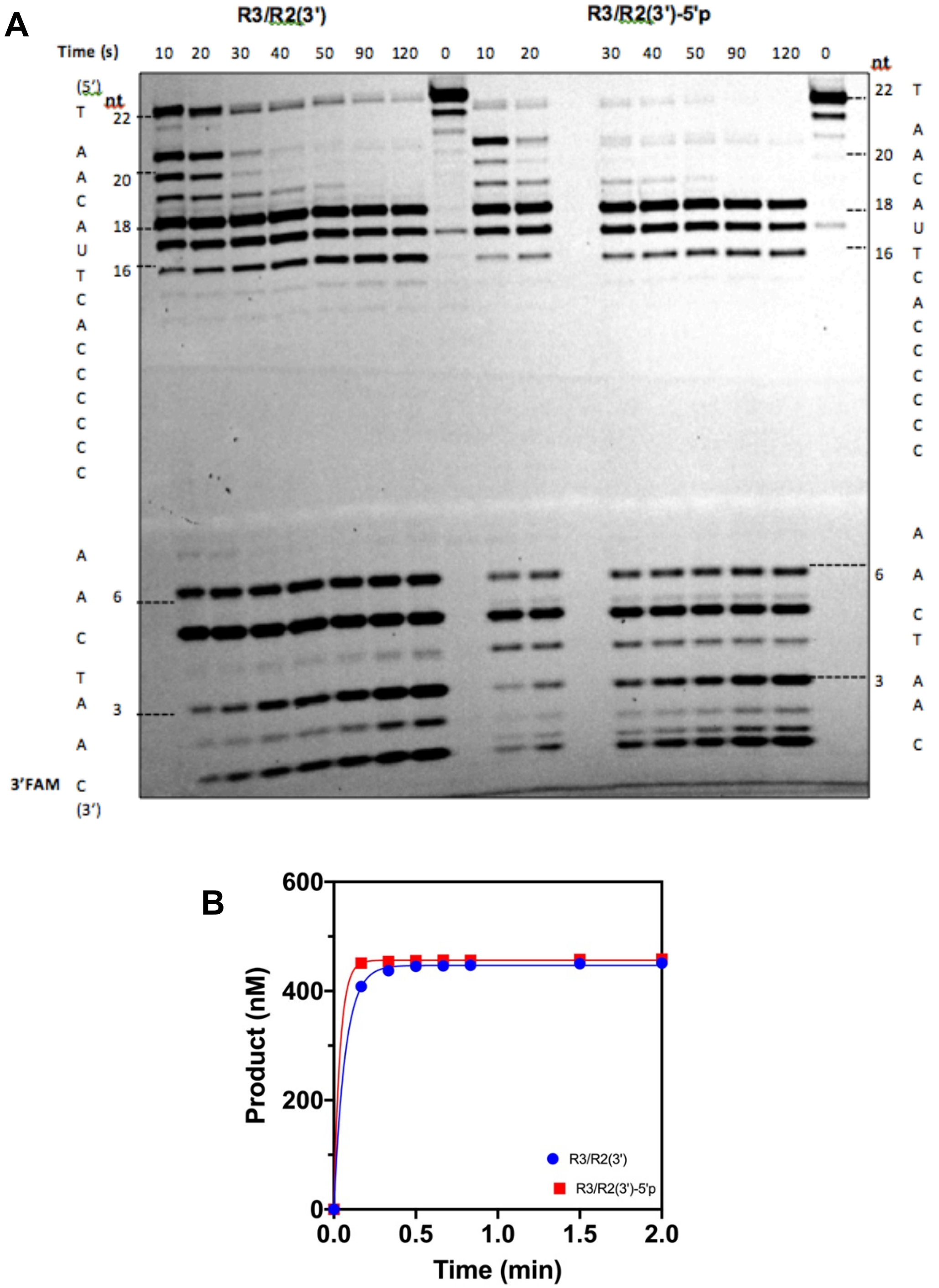

Figure S7. Single turnover assays with homopolymeric substrates, A20(5′), C20(5′), and T20(5′). Assays contained 4000 nM MGME1 and 1000 nM ssDNA. Samples were analyzed with 18% denaturing PAGE.

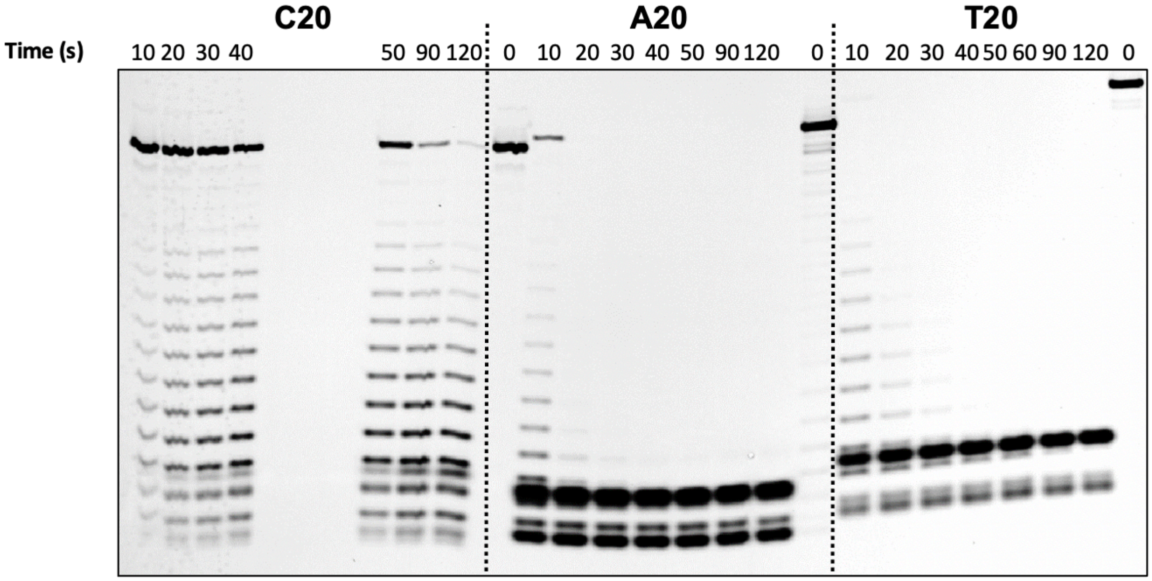

Supplement: Supplemental Figures S1–S7 and Table S1 [file mmc1.pdf]
